# Nanomaterials and Their Impact on the Immune System

**DOI:** 10.3390/ijms24032008

**Published:** 2023-01-19

**Authors:** Alaa A. Aljabali, Mohammad A. Obeid, Rasha M. Bashatwah, Ángel Serrano-Aroca, Vijay Mishra, Yachana Mishra, Mohamed El-Tanani, Altijana Hromić-Jahjefendić, Deepak N. Kapoor, Rohit Goyal, Gowhar A. Naikoo, Murtaza M. Tambuwala

**Affiliations:** 1 Faculty of Pharmacy, Department of Pharmaceutics and Pharmaceutical Technology, Yarmouk University, P.O. Box 566, Irbid 21163, Jordan; 2 Biomaterials and Bioengineering Lab., Centro de Investigación Traslacional San Alberto Magno, Universidad Católica de Valencia, San Vicente Mártir, 46001 Valencia, Spain; 3 School of Pharmaceutical Sciences, Lovely Professional University, Phagwara 144411, Punjab, India; 4 Department of Zoology, School of Bioengineering and Bioscience, Lovely Professional University, Phagwara 144411, Punjab, India; 5 Pharmacological and Diagnostic Research Centre, Faculty of Pharmacy, Al-Ahliyya Amman University, Amman 19328, Jordan; 6 Department of Genetics and Bioengineering, Faculty of Engineering and Natural Sciences, International University of Sarajevo, Hrasnicka Cesta 15, 71000 Sarajevo, Bosnia and Herzegovina; 7 School of Pharmaceutical Sciences, Shoolini University of Biotechnology and Management Sciences, Solan 173229, Himachal Pradesh, India; 8 Department of Mathematics and Sciences, College of Arts and Applied Sciences, Dhofar University, Salalah PC 211, Oman; 9 Lincoln Medical School, University of Lincoln, Brayford Pool Campus, Lincoln LN6 7TS, UK

**Keywords:** cytotoxicity, oxidative stress, reactive oxygen species, nanomaterials, immune system

## Abstract

Nanomaterials have been the focus of intensive development and research in the medical and industrial sectors over the past several decades. Some studies have found that these compounds can have a detrimental impact on living organisms, including their cellular components. Despite the obvious advantages of using nanomaterials in a wide range of applications, there is sometimes skepticism caused by the lack of substantial proof that evaluates potential toxicities. The interactions of nanoparticles (NPs) with cells of the immune system and their biomolecule pathways are an area of interest for researchers. It is possible to modify NPs so that they are not recognized by the immune system or so that they suppress or stimulate the immune system in a targeted manner. In this review, we look at the literature on nanomaterials for immunostimulation and immunosuppression and their impact on how changing the physicochemical features of the particles could alter their interactions with immune cells for the better or for the worse (immunotoxicity). We also look into whether the NPs have a unique or unexpected (but desired) effect on the immune system, and whether the surface grafting of polymers or surface coatings makes stealth nanomaterials that the immune system cannot find and get rid of.

## 1. Introduction

Our immune system has two main subsystems: the innate immune system and the adaptive immune system. The innate immune system is the first line of defense against foreign particles. It destroys infected cells or foreign material when it encounters them. This early, fast reaction is called the non-specific immunological response of the innate immune system. The innate immune system is fundamentally controlled by phagocytic cells (macrophages, dendritic (DCs), neutrophils, and mast cells (MCs)). The adaptive immune system works through specific cells, including T and B cells. Phagocytic cells, including antigen-presenting cells (APCs), destroy foreign antigens (bacteria, fungi, non-self-particles, etc.). T lymphocytes can also be divided into helper T cells (Th), which have subtypes called Th1, Th2, Th17, regulatory T cells (Tregs), and Th22 cells. Each subtype has owned its way to getting an immunological response from cells. For example, immune responses caused by Th1 cells are pro-inflammatory and involve cytokines such as interferon IFN-, interleukin (IL)-2, and tumor necrosis factor (TNF-α and TNF-β). Th2 cells are responsible for mediating a cellular anti-inflammatory antagonistic immune response [1]. 

Nanoparticles (NPs) can have positive or negative health impacts depending on their physical makeup, making them a “double-edged sword”. Physical-chemical properties, such as structural composition, surface charge, shape, crystallinity, surface area, zeta potential (surface charge), solubility, and surface functionalities, affect the NP toxicity. NPs should not be regarded as a homogeneous population with simple toxic properties because they independently mediate different biological reactions. Schrand et al. recently reviewed and outlined the toxicities of several metal-based nanoparticles, both in vitro and in vivo [2]. Although some nanomaterials are immunotoxins or immunomodulators, it would still be useful and vital for researchers to provide a concise overview of how NPs and the immune system interact. This review discusses how the innate and adaptive immune systems interact with different types of NP [3,4].

## 2. NPs and the Immune System

The immune system’s primary function is to recognize and eliminate foreign agents. An inadequate immune system affects the quality of life. There are several ways in which immunity must be improved or toned to suppress pathogenic agents and help with autoimmune problems [5]. Innate immunity is the non-specific and primary line of defense in the body that relies on pattern recognition receptors (PRPs) to identify large molecular patterns present in pathogens and pathogen-associated molecular patterns (PAMPs) and is composed of various components, including serum protein, cells, and physical obstacles, which work together to defend against invaders [6]. The innate mechanisms of the immune system and its protection from foreign entities make it more responsive to the absorption of phagocytic cells. The inflammatory response is another critical component of the innate immune system [7].

Adaptive immunity can also be divided into humoral immunity and cell-based immunity. The former is responsible for antibody-mediated reactions, which analyze and neutralize their targets, whilst the latter oversees cytotoxic T cell-mediated direct killing of disease-affected endogenous cells (antigen-specific). Simultaneous stimulation of PAMP through the pattern recognition receptor promotes co-stimulatory factor expression, a favorable sign of antigen-specific T cells in concert with the representation of the corresponding peptide-MHC representation [8]. Although antigen fragments can only be found in all cells via class I MHC (MHC-I) molecules, antigens found only in qualified APC cells can be presented through class II molecules, which are required for humoral immunity and T-helper cell proliferation [8,9,10]. These product degradations are then transported to the endoplasmic reticulum and then presented on the cell’s surface to form complexes using MHC-Class I products. If the virus gene protein or other intracellular proteins are formed by cytoplasm, fragments of this protein are also expressed on the cell’s surface in complexes with MHC gene products of class I T cell receptors and are then identified as foreign molecules, and T-cells (CD8-positive) with receptors are triggered and kill target cells with the external antigen [11].

**Figure 1:**
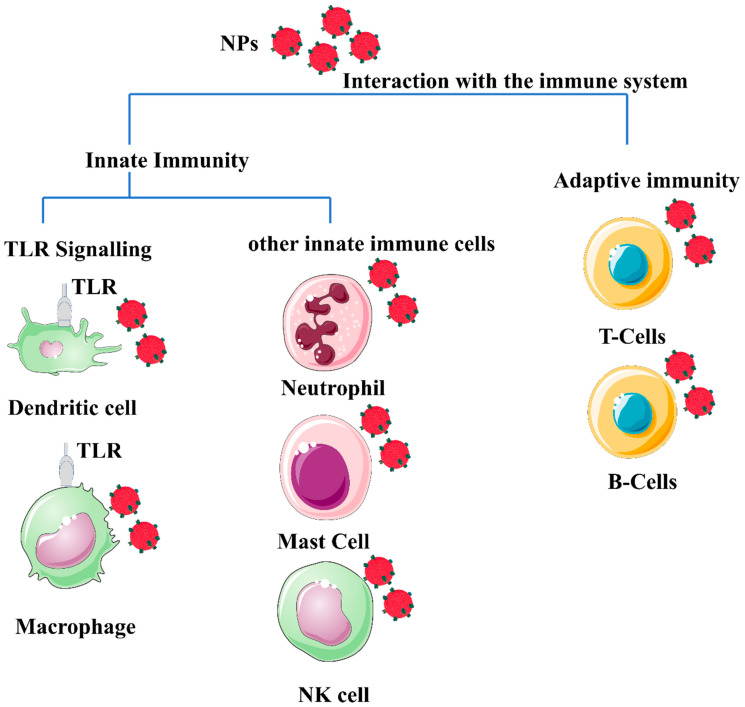
Schematic illustration of a generic form of nanomaterials (polymeric or metal-based) depicted in red spheres, showing their potential interaction with different immune system cells. The different properties of nanoparticles (NP) properties, such as morphology, size, surface charge, and composition, influence the interaction with immune cells. Most NPs induce an innate immune response. Mature innate immune cells such as lymphocytes, dendritic cells, monocytes, mast cells, neutrophils, macrophages, and natural killer cells, as well as pattern recognition receptors such as toll-like receptors (TLRs). In addition, nanomaterials’ interaction with the adaptive immune system activates Th1/Th2 responses and stimulates the production of cytokines. The effect of nanomaterials on B cells appears to enhance immunity during vaccination, and B cells are normally targeted by specifically functionalized nanomaterials to target B cells with lymphoma to kill lymphoma and generate a chemotherapeutic effect from the use of the NPs. The image was generated by BioRender.

The antigen presentation process is central to adaptive immunity, providing the material required to control downstream immune effectors. APCs, including dendritic cells, are responsible for scavenging and eliminating foreign materials [12]. Excessive stimulation of immune reactions is undesirable and will have negative consequences. Therefore, the influence of the immune system must be considered when designing a nanomaterial for in vivo applications, as shown in Figure 1.

Once a nanomaterial is designed for use in vivo, three immune-related effects should be considered. The first is degradation or rejection through the immune system, which may result in a protective immune response. The second point is immunotoxicity, which could damage and trigger inflammatory and pathological changes in the immune system. Third, immune compatibilities do not affect the immune response [3,13].

## 3. Nonspecific Immunomodulation

NPs were used as a cytokine delivery tool, helped modulate physical localization, and served as a repository for sustainable release. The justification was to help effectively manage the nontarget cytotoxicity associated with these treatments.

### 3.1. Nanomaterials and Immune System Modulations

The sheer quantity of physicochemical properties of NPs, including shape, size, morphology, and elemental constituents, makes the investigation of their cytotoxicity consequences complex and challenging. Oxidative stress, inflammation, genetic damage, cell division, and cell death suppression are paradigms for NP-mediated toxicity. Considerable existing research has revealed that NP cytotoxicity is commonly associated with ROS production (which may be either protective or harmful during biochemical processes), and subsequently, oxidative stress is frequently reported with NPs toxicity [14]. ROS includes superoxide radicals (O_2_^•^), hydrogen peroxide (H_2_O_2_), hydroxyl radicals (^•^OH), and singlet oxygen (^1^O_2_). Biological systems produce them as metabolic by-products. Adequate oxidative stress is the foundation for many other activities, including induction of protein phosphorylation, multiple transcription factor activation, cell apoptosis, host defense, and differentiation [15]. Intrinsically or externally, ROS can be synthesized inside the cell. Molecular oxygen is generated by O_2_^•^, and primary ROS is mediated by nicotinamide adenine dinucleotide phosphate (NADPH) oxidase through a single electron decrease. Using a metal to speed up the Fenton reaction, a further reduction in oxygen could change H_2_O_2_ or ^•^OH into something else [16,17].

Inflammatory phagocytes, such as neutrophils and macrophages, generate oxidative outbursts as a defensive mechanism against environmental contaminants, tumor cells, and microorganisms. Intracellular calcium levels activate transcription factors and modulate cytokine synthesis by mechanisms of free radicals and therefore contribute to the influence of NP on oxidative stress [18]. Glutathione-S-transferase (GST) is a phase II enzyme family that catalyzes electrophilic detoxification and protects cells from mutagens, carcinogens, and glutathione, all of which act as free radical scavengers. GST in the cytosol eliminates H_2_O_2_. GST interacts with H_2_O_2_ in a glutathione peroxidase catalyzed chemical reaction that results in glutathione disulfide (GSSG). Glutathione reductase (GR) catalyzes GSSG glutathione regeneration using the NADPH hexose monophosphate shunt (HMPS) produced by NADPH [19].

NP-mediated ROS responses orchestrate a cascade of adverse pathological processes such as inflammation, genotoxicity, fibrosis, and carcinogenesis. Nanomaterials with different chemical compositions, such as fullerenes, CNTs, and metal oxides, have been reported to promote ROS. The major variables in NP-induced ROS are (i) active redox cycling on the surface of NP due to transition metal-based NP, (ii) surface functional groups, and (iii) NPs–cell interactions [20]. Furthermore, CNT-induced oxidative stress, for example, promotes cellular signaling pathways, increasing the production of pro-inflammatory cytokines. Some NPs have been shown to activate inflammatory cells, such as macrophages and neutrophils, increasing ROS production. Several NPs, such as titanium dioxide (TiO_2_), zinc oxide (ZnO), cerium oxide (CeO_2_) and silver NP, have been documented to deposit on the cellular surface or even inside intracellular organelles and trigger oxidative stress signaling cascades, resulting in oxidative damage to the cell [21]. Because of their surface characteristics, NPs, such as Si and Zn with identical particle sizes and shapes, exhibit varied cytotoxicity responses. Since ZnO is more chemically active than SiO_2_, it generates more free radicals, leading to increased oxidative stress [22]. The process of ROS formation differs for each NP, and the precise underlying cellular mechanism for ROS generation is still unknown and must be explored [23].

### 3.2. Immunological Effects of Nanomaterials (Immunotoxicology)

When used in vivo, NPs and the interface between the NP and the biological material play a key role in how the nanomaterial is transported, eliminated, and deposited, especially when it comes to the systemic administration of medicines. A protein crown effect, also known as a protein corona particle, is formed when NPs encounter biofluids for the first time. The way in which NPs interact with plasma proteins and other biomolecules changes their bioactivity, which in turn causes several changes in the way the body works. When the immune system encounters a foreign body, such as NPs, the first cells to respond are phagocytic cells. Several conditions, including inflammation, are caused by overactive immune systems, whereas immunosuppression leaves the host susceptible to infections by the inhalation of CNTs was used to decrease B cell activity via NP involvement in immunosuppression [24]. The alveolar macrophage produced is important for the immunosuppressive process observed. When the immune system encounters a foreign body, such as NPSs, the first cells to respond are phagocytic cells. There have been several reports of negative interactions between the immune system and nanoparticles, with immune stimulator immunosuppression potentially leading to inflammatory or autoimmune diseases and increasing the receptor likelihood that the body will get infected. Immunosuppressive NPs were used to reduce B cell activity by allowing people to breathe in CNTs. However, when a nanomaterial is administered subcutaneously or intradermally, stimulation of the complement system by NPs can improve the effectiveness of the treatment. Modifying the physicochemical features of NPs allows for control over the pathways by which they activate the complement system. After being exposed to nanoparticles, important biological processes happen, and mast cells play a role in inflammation and the toxicity of some NPs [25,26].

### 3.3. Mechanism of Nanomaterials Toxicity

**Figure 2:**
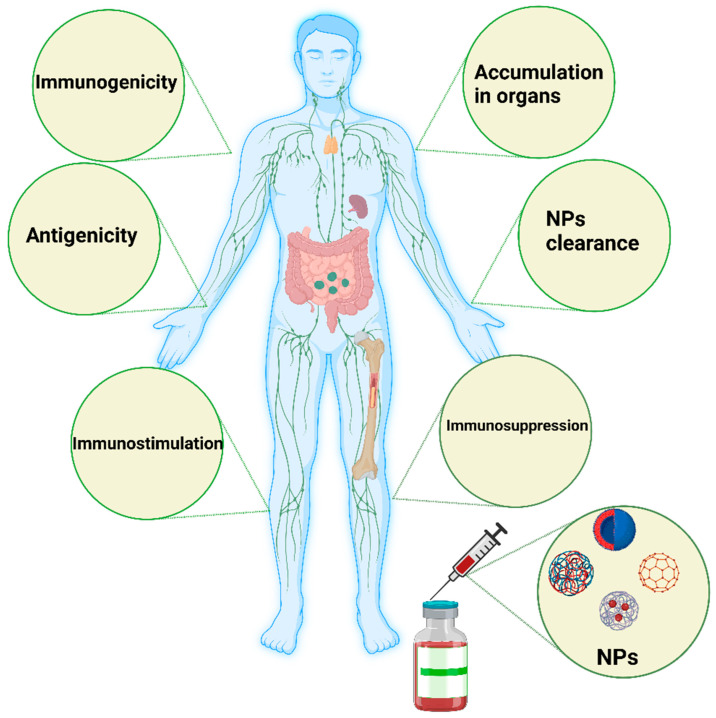
Schematic illustration of the major influence of nanomaterials on the immune system. Nanomaterials can induce immunogenicity, which is the ability of NPs to induce humoral or cell-mediated immune responses, whereas the antigenicity effect of NPs deals with the ability of such materials to react with antibodies or T cells. NPs could be engineered to avoid interactions with the immune system or specifically interact with the immune system to simulate or suppress their effects. In some cases, the immune system stimulation generated by such NPs could manifest as a hypersensitivity reaction, anaphylactic shock, or inflammation. In immunosuppression, NPs can have a stimulating effect on immune system components, such as APCs, B cells, and T cells, directly or indirectly. The degradation and clearance of NPs are the main factors contributing to the use of such materials in clinical settings. Tiny NPs below 10 nm are rapidly excreted by the kidney and liver. Particles larger than 100 nm can be eliminated by the mononuclear phagocytic system, such as the lymph nodes and spleen. The image was generated by BioRender.

Direct or indirect modulation of oxidative enzymatic reactions by NPs causes oxidative stress because of free radicals, or ROS, such as superoxide anions and hydroxyl radicals. Multiple factors contribute to oxidative stress in the body. Cellular responses to nanoparticles are accompanied by the production of ROS, which can be stimulated by the oxidant characteristics of the particles themselves. Free radical intermediates are present on the reactive surfaces of particles and are quite stable; however, there are effects of NP functionalities on the formation of redox-active groups. A summary of the influence (immunogenicity, antigenicity, clearance, immunostimulation, and immunosuppression) of nanomaterials on the immune system is shown in Figure 2.

### 3.4. Nanomaterials Affect Cell Functions

The practical problem of immunosuppression and immunostimulants mediated by NPs is highly innovative, as the interaction of NPs with the body’s immune system and their impact have not been fully understood; nevertheless, there is a considerable amount of evidence that illustrates the enormous potential of these immunomodulation strategies for the treatment of immunological disorders. The use of NPs and the modulation of immunostimulants and immunosuppression are summarized in Table 1.

### 3.5. Immunosuppression

Immunosuppression occurs when a medication or other substance inhibits the activation or effectiveness. Immunosuppression is commonly thought to be an immunotoxic impact linked to various human diseases. The size, morphology, and composition facilitate blood, cellular, and protein adsorption, allowing for more efficient interactions with immune cells and the subsequent immunological response. AuNPs are an excellent example of these effects. Larger AuNPs and rod-shaped geometries are routinely internalized via sophisticated uptake mechanisms. Spherical NPs between 5 and 30 nm in diameter are susceptible to passive association with cells. The surface coating does have an impact on their absorption by cells. For example, citrate or lipid capping of the AuNPs may increase the particles’ stability and passive intake [33,34]. On the other hand, since silver and gold NPs interact with adaptive and innate immune systems, few studies have elucidated the mechanism behind the potential of noble metal NPs to induce an immunosuppressive response [35]. Citrate-coated AuNPs have been shown to have no measurable cellular or organ toxicity in mice. However, researchers reported anti-inflammatory potential that reduced cellular responses triggered by interleukin 1 beta (IL-1β). IL-1 β is an inflammatory cytokine that mediates adaptive immune responses, as well as common inflammatory conditions such as rheumatoid arthritis [36,37]. The potential of monodispersed citrate-coated AuNPs varying in size from 5 to 35 nm to regulate pro-inflammatory functionality induced by IL-1β generation was examined. The IL-1β pathway disrupted the smallest nanoparticles, measuring 5 nm. Larger nanoparticles (>10 nm) had less of an impact, while 35 nm particles did not affect the IL-1 pathway [38]. Furthermore, poly(acrylic acid)-coated AuNPs have been shown to have opposite effects in the same THP-1 cell lines by promoting inflammation [39].

Immunosuppression produced by AgNPs has received less attention than AuNPs. Silver nanoparticles stimulated the production of TNF-, IL-6, IL-8, IL-1, and IL-11 production [40]. Tian et al. demonstrated that topical treatment of AgNPs modulated cytokines at a wound site. IL-6 mRNA expression was considerably reduced during the healing phase, whereas TGF-1 expression increased. It has also been demonstrated that local and systemic applications can reduce inflammation [41]. In addition, iron oxide NPs have been shown to induce immunosuppression and anti-inflammatory activities. Ovalbumin-sensitized mice received ovalbumin, a T cell-dependent antigen, after an administration of iron oxide nanoparticles in investigations by Liao et al. and Shen et al. Antigen-specific antibody expression, as well as antigen-specific cytokine secretion in the spleen, was considerably decreased. Iron oxide nanoparticles inhibited the activity of T helper cells and macrophages while decreasing interferon, IL-6, and TNF-expression [42,43]. TNF- and IL-6 are both cytokines that contribute to inflammatory responses. Iron oxide NPs also changed the proportion of helper T cells, suppressing the allergic response. In an alternative animal model, administration of iron oxide particles preceding immunological activation with an endotoxin lowered IL-1 expression in microglia cells. Iron oxide NPs hindered cytokine processing pathways, culminating in the attenuation of IL-1. Human dendritic cells treated with poly(vinyl alcohol)-coated iron oxide NPs exhibited impaired antigen processing and T cell activation in a separate study. Injections of iron oxide nanoparticles were given again, and the inflammation was reduced [44]. According to Jan et al. [45], iron oxide NPs accumulated in lysosomes, improving lysosomal accessibility, and reducing cathepsin B activity.

**Figure 3:**
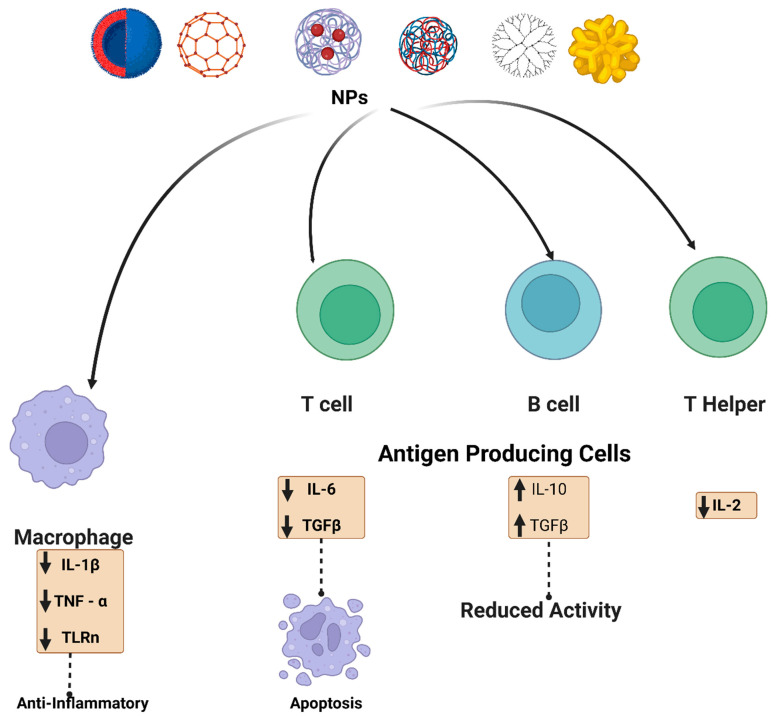
Schematic illustration of the NPs interaction and effect on the immune system. NPs (metalic, polymeric, dendrimer, carbon-based and lipid-based) interact with immune cells such as antigen-presenting cells, B cells, T cells, and macrophages. For example, carbon-based nanomaterials are involved in up-regulation of transforming growth factor (TGF-β), interleukin-10 (IL-10), and it also decreased B cell activity in addition to apoptosis. Metal oxide NPs have also been reported to affect adaptive immune cells, such as cerium oxide NPs, which are responsible for scavenging reactive oxygen species (ROS). Polymeric NPs and dendrimers exhibit an assortment of immunosuppressive effects. Some possible mechanisms by which various nanoscale compounds might dampen the immune system are illustrated.

Furthermore, cerium can consistently switch redox potential between Ce^4+^ and Ce^3+^, allowing cerium oxide NPs to absorb reactive oxygen species efficiently. As cerium oxide switches between oxidation states, it creates oxygen vacancies in the crystal lattice. Since the vacancies are clustered at the NPs’ surfaces, cerium oxide NPs have a high capability to interact with free radicals immediately. This antioxidant potential has been demonstrated in several studies [45]. Cerium oxide NPs antioxidant properties allow them to suppress inflammation caused by nitric oxide synthase production [46]. Superoxide dismutase-2, an oxidative stress mediator, was also upregulated by cerium oxide NPs. The antioxidant properties of cerium oxide NPs, similar to those of many other NPs, are influenced by their size and morphology. As a result, smaller cerium oxide NPs outperformed their larger counterparts in antioxidant capacity. Figure 3 summarizes the various effects of the NPs on different immune cells, depending on whether immunosuppressive or inflammatory cytokines are secreted.

Polymer NPs and macromolecules (dendrimers) exhibit an assortment of immunosuppressive effects. Some possible mechanisms by which various nanoscale compounds might dampen the immune system are illustrated. Fullerenes (C60), such as cerium oxide NPs, efficiently reduce oxidative stress by lowering ROS levels. Compared to cerium oxide NPs, fullerenes disseminate reactive oxygen species via their aromatic structure. Fullerenes reduce both hydroxyl and superoxide oxygen radicals, and many of their free radical-scavenging abilities have been regulated by bioconjugation of fullerenes with water-soluble ligands [47,48]. Fullerenes have been reported in vitro and in vivo to suppress oxidative stress, even though the immune response is strongly dependent on administration and dosing. The administration of low doses of fullerenes by intraperitoneal administration or inhalation significantly reduced oxidative stress [49]. Hydroxylated fullerenes have been used to protect RAW 264.6 cells against oxidative stress in vitro and ischemia-perfused lungs in rats. Fullerenes can inhibit the release of human mast cells and peripheral blood basophils by reducing the signaling pathways associated with oxygen radical quantities [50]. Basophils and mast cells are essential in inflammatory responses, including type I hypersensitivity reactions. Type I hypersensitivity responses are usually triggered by persistent exposure to the allergen. B cells produce allergen-specific immunoglobulin E (IgE), which binds to receptors and sensitizes mast cells or basophils upon first allergen exposure. Following exposure to the allergen, IgE crosslinks and mediators, including prostaglandins and histamine, are activated. Basophils and mast cells lowered IgE signaling and ROS formation following fullerene administration, preventing the release of histamine [51].

Furthermore, Mitchell et al. revealed that inhaling multi-walled carbon nanotubes (MWCNTs) in tiny amounts stimulated T-cell dysfunction pathways that suppressed the immunological response from splenic lymphocytes, but there was no toxicity or immunosuppressive action in the lungs. Exposure to MWCNT significantly reduced natural killer cellular activities, increased prostaglandin synthesis, and increased IL-10 production [52]. In contrast, single-walled carbon nanotubes (SWCNTs) have been shown to repress immune system mediators in human lung epithelial cells. Additional investigations found that inhalation of SNCNTs increased lung inflammatory responses and decreased T cell responsiveness after exposure in mouse models; this immunosuppression seemed related to the direct effects on dendritic cells [53]. The observed differences in immunological response between fullerenes, MWCNTs, and SWCNTs may be attributed to their complex geometries and electrochemical properties. Research to unravel these effects in the future could contribute to a better understanding of the implications of surface charge and intrinsic conductivity on the immune system response [54,55].

Polystyrene latexes are one form of polymeric NPs that are studied for an immunological response. Polystyrene latexes were shown to produce immunosuppressive and immunostimulatory impacts. Polystyrene NP suppressed lung inflammation after allergen exposure, which was ascribed to inhibition of dendritic cell expansion within the lungs [50]. Another investigation employing polystyrene nanoparticles highlighted the significant impact of surface charge on the immune response. By coating polystyrene nanoparticles with charged sulfonate and phosphonate groups, Frick et al. altered their surface properties. This caused dendritic cell maturation and increased CD4+ T cell activity, resulting in immunostimulant activity [56]. Finally, by inactivating pathogenic T cells, antigen-decorated polystyrene NPs induced T-cell tolerance and suppressed autoimmune encephalomyelitis. This considerable effect of surface groups on the immunological response of basic polystyrene nanoparticles emphasizes the need for a comprehensive study of the immunology of polymer nanoparticles, which are often commended or promoted for the delivery of drugs and other nanomedicine purposes [57].

The anti-inflammatory activities of polyamidoamine dendrimers were established by chance while studying their potential as a drug delivery system. Polyamidoamine dendrimers with amine or hydroxyl surface functional groups significantly suppressed pro-inflammatory activities. Dendrimers with carboxylate surface groups had no significant anti-inflammatory effects. In microglia cells, hydroxyl-terminus polyamidoamine dendrimers also inhibited the secretion of pro-inflammatory regulators such as nitric oxide and IL-6 [58]. Hayder et al. likewise utilized anionic azabisphosphonate moieties to alter the surface charge of dendrimers. These anionic dendrimers were administered to arthritic mouse models and suppressed the release of pro-inflammatory cytokines and the osteoclastogenesis pathway [59].

Further, polyamidoamine-glucosamine dendrimers have been proven to block TLR-mediated inflammatory responses and diminish IL-6 and IL-8 release [60]. Imidazoquinoline-based dendrimers with imidazoquinoline as the core ingredient rather than glucosamine are analogous to polyamidoamine-glucosamine dendrimers. Because imidazoquinoline is a TLR7 and TLR8 agonist, imidazoquinoline-based dendrimers inhibit TLR7 and TLR8 activities [61]. This shows that the surface charge is pretty simple and that changing the chemistry of the surface could be used to make NPs that precisely modulate the innate immune response. 

Liposomes can also be utilized to improve the localization of immunosuppressant drugs that have been entrapped. Hong et al. and others investigated the effectiveness of encapsulating IL-10 genes in cationic liposomes to enhance allograft survival after a heart transplant. Liposome administration led to local overexpression of IL-10 and a decline in lymphocyte reactivity. Similarly, after a liver transplant, canines received liposomal tacrolimus, an immunosuppressive treatment, and survived substantially longer than dogs who received tacrolimus intravenously [62,63]. When used to treat rheumatoid arthritis in rats, glucocorticoids incorporated within liposomes result in much less cytotoxicity and enhanced inhibition of anti-inflammatory cytokines than free drug administration [64,65].

### 3.6. Immunostimulant

The propensity of NPs to induce innate or adaptive immune responses is being used to assess their immunostimulatory potential as summarize in Table 2. The stimulation of the complement cascade can be detrimental if particles penetrate the systemic circulation accidentally or on purpose, resulting in hypersensitivity responses and anaphylaxis [66]. The size has been proposed to be an important factor in determining whether antigens loaded into nanoparticles produce type I (interferon-) or type II (IL-4) cytokines, thus contributing to the type of stimulatory immune response [67].

Induction of allergic responses is one aspect of NP-mediated immunostimulation. A few investigations have connected NP exposure to allergy responses in test animals and humans. In mice, for example, SWCNT and MWCNTs equally increased the allergenicity of egg albumin, whether administered by intranasal or subcutaneous injection routes. The CNT-mediated activation of the acute inflammatory response is hypothesized to be the mechanism underlying this enhanced allergenicity [68].

In rare circumstances, nanoformulation of an authorized treatment would prevent allergic responses linked to previously approved preparations, and abraxane is an illustration of this reformulation. In this instance, reconstituted paclitaxel-bound albumin nanoparticles provoked no allergic response, but the first-generation formulations of paclitaxel in the non-ionic surfactant Cremophor EL triggered significant hypersensitivity, commonly involving premedication with only steroids and a histamine blocker [69]. Almost all the immunostimulatory responses elicited by nanoparticles are mediated by the release of pro-inflammatory cytokines. Numerous studies have provided evidence through various forms of nanomaterials that can induce cytokine production, i.e., AuNPs, lipid-based NPs, dendrimers, etc. [70,71,72].

The diameter of NPs has been perceived as a determinant factor in influencing an NP’s ability to generate cytokine activation. For example, CNT length has been reported to correlate with in vivo subcutaneous injection inflammation caused by CNT administration [73]. On the contrary, some studies have demonstrated that surfactants or microbial endotoxins included in the formulations, rather than NPs, trigger cytokine production. As a result, when analyzing the inflammatory properties of NPs, it is vital to evaluate the levels of chemical (byproduct generation) and biological (endotoxin) components.

**Table 2:** NP-based immunostimulatory effect and study results from the studies outcome.

Type of NPs	Study Outcomes	References
Liposomes decorated with synthetic long peptides antigen	The immunological response was mediated by antigen-specific CD8^+^ T cells that were induced	[74]
Liposomes	Induction of antigen-specific response	[75]
Liposomes loaded with cytosine-phosphate-guanine and 3,5- didodecyloxybenzamidine	DCs were stimulated to release cytokines, co-stimulatory molecules were expressed, and an antigen-specific immune response was enhanced	[76]
AuNPs	Macrophage activation	[77]
AuNPs loaded with BSA antigen	Anti-BSA antibodies were detected in greater concentrations in the blood serum of mice inoculated with BSA–AuNPs and cytosine-phosphate-guanine –AuNPs conjugates	[78]
MWCNTs	Inducing strong CD4^+^ T and CD8^+^ T- cells mediated immune response	[79]
MWCNTs loaded with anti-CD40 Ig	In subcutaneous or lung pseudo-metastatic tumor models, it increased entrapped ovalbumin-specific T cell responses and suppressed the development of entrapped ovalbumin–expressing B16F10 melanoma cells.	[80]
MWCNTs loaded with ovalbumin	Elicited a strong anti-tumor immune response	[81]
MWCNTs loaded with cytosine-phosphate-guanine	Elicited a strong cellular and humoral immune response	[82]
Iron oxide (Fe_3_O_4_) NPs	In vitro, it induced a significant adaptive immune response by stimulating DCs and macrophages, and it reduced tumor development and prevented tumor formation in vivo	[83]
Iron oxide (Fe_3_O_4_) NPs	Enhanced T cell activation and elevated stimulation of anti-tumor activity	[84]
Micelles loaded with cytosine-phosphate-guanine and Trp2	In tumor-bearing mice, it produced antigen-specific cytotoxic CD8^+^ T cell-mediated immunity, as well as a robust anti-cancer immune response	[85]
Micelles loaded with cytosine-phosphate-guanine and Trp2	Trp2/PHM10/ cytosine-phosphate-guanine nanoformulation dramatically increased CD8^+^ T cellular activities while improving anti-tumor effectiveness.	[86]
Micelles loaded with ovalbumin and CL264 agonist	A robust antigen-specific cellular and humoral immune response was elicited	[87]
Dendrimers loaded with ovalbumin and cytosine-phosphate-guanine	Elicited a much greater T-cell-mediated immunological response	[88,89]
Dendrimers loaded with cytosine-phosphate-guanine	Cytosine-phosphate-guanine delivered effectively into DCs triggered an adaptive cellular immune response	[90]
Protein NP loaded with melanoma-associated gp100 epitope and cytosine-phosphate-guanine	Antigen-specific antitumor immune response substantially increased	[91]
NP protein loaded with peptide epitope cytosine-phosphate-guanine	Elevated CD8+ T cell activation and antigen cross-presentation	[92]

### 3.7. Are Nanomaterials Immunogenic?

Macfarlane Burnet proposed the idea of “clonal selection,”, which states that B-cells sensitive to a given antigen already exist in the organisms well before the antigen is encountered. Although not all antigens can stimulate an immune response. The immunological response to an antigen is determined by characteristics inherent in the antigen itself, such as its source, structural composition, shape, size, and the inclusion of repeating epitopes. In many cases, additional stimulation beyond the presence is necessary for antibody production to be effective. When a B-cell recognizes its corresponding antigen and binds to it, a cascade of events begins that ultimately leads to the maturation and differentiation of the cell into plasma cells, which generate antibodies [93]. The thymus-dependent (TD) and thymus-independent (TI) pathways contribute to antibody production. Proteins often initiate the TD process, which begins with DCs taking in antigen and being activated. DCs are APCs that release cytokines that activate T-helper cells that are competent to recognize antigen presented in the context of the major histocompatibility complex (MHC II). Subsequently, activated T cells communicate with B cells, which deliver the corresponding antigen in the context of MHC II. When T-helper cells interact with B-cells, the B-cells multiply and differentiate into plasma cells. Immunological memory, high-affinity antibody production, and isotype switching are hallmarks of the TD route [94].

Most NPs are either not immunogenic or just weakly provoke an immune response because of their relatively small size. Because of this, it is often claimed that immunizing animals with NPs, even in the presence of potent adjuvants, does not induce the production of antibodies that target the nanoparticles [95]. For example, in the presence of Freund’s adjuvant, vaccinating rabbits using fullerene analogues did not result in the development of fullerene-specific antibodies [94]. Moreover, when the fullerene analogues were tethered to thyroglobulin as a protein carrier and used in the vaccination process, antibodies specific for the C60 fullerene were produced [96]. Table 3 summarizes the nanomaterials discussed in this review, their composition, formulation, and their medical uses.

### 3.8. NPs and the Effect of Toll-like Receptors

When entering the body, NPs are identified by the immune system and will have the ability to inhibit or stimulate an immune response. However, non-toxic NPs can alter a normal defensive response towards TLR ligands. TLRs are expressed in dendritic cells and play a crucial role in innate and adaptive immunity by sensing intracellular and extracellular pathogens. They trigger the activation of biological systems that result in increased activity of T and B cells and macrophages [158]. TLRs can be stimulated by metallic NPs, according to recent research. TiO_2_, ZnO, ZrO_2_ and AgNPs, particularly, alter immunological responses via TLRs [159,160,161]. NPs can trigger cytokine production and pro-inflammatory responses. For example, 25 μg/mL for TiO_2_, 30 μg/mL for Fe_3_O_4_, 1 μg/mL ZnO, 15 μg/mL for Ag_2_O and 0.5 μg/mL for CuO were used to estimate the influence of such NPs on the expression levels of the TLR-4 and TLR-6 mRNAs in THP-1 monocytes. The study reports that all listed NPs led to an increase in TLRs expression levels to different degrees based on the type of the NP. The CuO, ZnO, and TiO_2_ NPs significantly enhanced expression: 1.2, 1.2, and 1.1 times, respectively. TLR-4 expression was unaffected by Ag_2_O or Fe_3_O_4_. Ag_2_O, TiO_2_, and CuO were more powerful than the other NPs, with fold induction values of 1.5, 1.5, and 1.5, respectively. ZnO and Fe_3_O_4_ NPs exhibited the least comparable impacts, increasing expression by 1.4-fold [162].

TLR agonists such as CpG ODN [163], Imiquimod [164], poly I: C [165], MPL [166] and PAM3CSK4 (TLR2 ligand) [167], have been adjuvanted to NPs that deliver antigens. Lipid-based NPs, metal-based NPs, and polymeric nanomaterials [168] were used to efficiently deliver drugs together with TLR ligands to stimulate the cell surface or intracellular TLRs or to reduce possible negative effects due to systemic delivery of free synthetic adjuvants. Immunization with TLR7/8 or TLR9 ligands, as well as ovalbumin (OVA)-encapsulating poly(lactic-co-glycolic) acid (PLGA) NPs, induces superior humoral and cellular immune responses with local immune activation, but reduces systemic inflammation [169]. Furthermore, Kim et al. demonstrated that TLR-7 and TLR-8 agonist encapsulation into PLGA NPs dramatically boosted the stimulatory expression of CD86, CD80, and CD40 compared to the untreated agonist via the stimulation of bone marrow-derived dendritic cells [170]. Furthermore, subcutaneous delivery of the nanoformulation enables it to migrate to a draining lymph node, where it stimulates DCs and CD8^+^ T cells (cytotoxic T cells), resulting in an enhanced anticancer response in bladder, melanoma, and renal carcinoma models, demonstrating the importance of PLGA NPs as effective immunostimulatory adjuvants for cancer immunotherapy [171].

## 4. NPs and Allergy

Researchers have hypothesized that up to four thousand different compounds might cause skin irritation. Because hypersensitivity to chemical substances may be caused at very low levels of exposure to that material through skin contact, for example, this could be a potentially significant nanotoxicity of nanomaterials. Park et al. demonstrated that topical skin treatment with amine-modified polystyrene NPs with a diameter of 50 nm or TiO_2_ NPs did not produce skin sensitization [172]. Mesoporous SiO_2_ and colloidal SiO_2_ NPs, both having dimensions of around 100 nm, were tested for their sensitization potential by Lee et al. Applying each type of NP to the skin for three days in a row resulted in only minor changes in the thickness of the ear skin. Using a local lymph node test, the researchers showed that neither NP caused skin sensitization. According to these findings, many nanomaterials may not significantly increase skin sensitivity when being topically applied to healthy skin. Because nanomaterials do not easily pass-through healthy skin, skin painting is an ineffective method for assessing the sensitivity potential of synthetic chemicals. As a result, if nanoparticles can pass through allergic or injured skin, subcutaneous or intradermal delivery may be appropriate for studying nanomaterial sensitivity [173]. According to epidemiological research, the presence of metal sensitizers in airborne particles has been linked to an increased risk of developing metal allergies. The sensitization propensity of both NPs and the metal ions they produce is important when dealing with metal NPs. The prevalence of metal allergy, a major causative agent of atopic dermatitis, is demonstrated by the fact that up to 17% of women are affected by it [174,175]. Activation of innate and adaptive immunity is hypothesized to be the mechanism by which metal ions from jewelry and clothing (buttons, zippers, and belt buckles) induce metal allergy. Metal allergies are thought to originate in metal-ion-induced T cells, which are reactive to metal ions in the major histocompatibility complex. However, several attempts to sensitize mice with simple metal-ion therapy have failed [174].

## 5. Interaction with the Immune System

Currently, different types of NPs are used to deliver many therapeutic agents to improve their therapeutic effects and/or reduce their unwanted side effects [176]. The rapid expansion of the use of nanoparticles in drug delivery and disease diagnosis has accompanied by an increase in research into the potential immune response of these nanomaterials. The used NPs might interact with phagocytes, lymphocytes, neutrophils, or other immune cells, resulting in various immune responses such as inflammation and hypersensitivity. This interaction with immune cells depends on the physicochemical properties of NPs, and this interaction can be modified by changing the specific properties of the NPs used, such as particle size, shape, hydrophobicity, stiffness, and their surface components [177].

Lipid-based NPs, polymeric NPs, metal-based NPs, silica NP, and carbon nanotubes are examples of NPs drug delivery systems. Among these types, lipid-based NPs, such as liposomes and niosomes, are among the most studied types of NPs [178]. With the use of these NPs delivery systems, several successes have been achieved with many NP-based formulations that have been translated to the market, such as Doxil and AmBisome, which are liposome-based formulations for the delivery of doxorubicin and amphotericin, respectively [179]. However, this rapid and successful increase in the use of these NPs as delivery systems is accompanied by limited knowledge about their anticipated toxicity and immunity. This is because of the different components that can be used to prepare these NPs. The different NPs’ uptake can be seen by the innate immune cells based on the particle size. The mechanisms of NP uptake include pinocytosis and micropinocytosis. Kruth et al. investigated the involvement of a pinocytosis mechanism for the uptake of their fluorescent PEGylated NPs by macrophages in vitro and in vivo models [27]. Sometimes, immune cells might uptake the NPs using different uptake mechanisms. For example, Gu et al. demonstrated the uptake of superparamagnetic iron oxide NPs with an average size of 10 nm by RAW 264.7 macrophage cells through the involvement of both macropinocytosis and caveolae-dependent endocytosis [180].

Furthermore, the uptake of AuNPs prepared by Oli et al. was size dependent, with 10 nm particles engulfed by clathrin/caveolar mediated endocytosis at a significantly higher rate than 50 nm particles [181]. Rothen et al. prepared polystyrene NPs in two sizes; 40 and 600 nm. Through the use of the macrophage model, 40 nm NPs were taken up through phagocytosis or micropinocytosis in addition to clathrin-mediated endocytosis, while 600-nm NPs were internalized by micropinocytosis and phagocytosis [182,183]. This difference in the mechanism of the uptake of NPs could result in different immune responses after NP uptake. When compared to the same NPs with an average size of 125 nm in an in vitro macrophage model, the use of hollow nanoparticles with an average size of 50 nm resulted in a significantly higher rate of induction of various inflammatory cytokines such as IL-1, IL-6, and TNF [184]. The NP surface will influence the level of induction of the immune system. Liu et al. created polymer-based NPs with varying degrees of hydrophobicity and discovered that increasing the hydrophobicity of NP increased cellular uptake and subsequent immune responses [185]. Moreover, the pro-inflammatory cytokine expression in splenocytes from mice was increased by using hydrophobic NPs with a cationic charge. Moreover, the pro-inflammatory cytokine expression in splenocytes from mice was increased by using hydrophobic NPs with a cationic charge [186]. In the work of Saha et al., the use of positively charged Au NPs was associated with a higher level of macrophage uptake in vitro due to the high rate of serum proteins binding to these NPs [187]. Liposomes are made up of bilayer phospholipids with the addition of cholesterol. Different types of natural and synthetic phospholipids can be used to prepare liposomes [179,188].

After liposome administration, the immune system binds to the opsonin protein through the opsonization process. This recognition will result in the rapid elimination of these liposomes through the reticuloendothelial system. This elimination was shown to be higher for charged liposomes compared to neutral ones [178]. To minimize the rate of liposome elimination, hydrophilic polymers such as polyethylene glycol (PEG) can be used on the outer surface of liposomes to act as a polymer coat, reducing their elimination rate [178]. Several studies have found that coating liposomes with PEG reduces liposome elimination. For example, Dadashzadeh et al. indicated that incorporating PEG with different liposome preparations with an average size of 100 nm improved peritoneal accumulation, particularly with negatively charged liposomes, after in vivo administration in a mouse model [189]. PEG-coated cationic liposomes prepared by Olivier et al. were shown to have enhanced stability of the prepared vesicles and enhanced accumulation of the oligodeoxyribonucleotide (ODN) payload inside these liposomes at the target site. This was proven by improving ODN uptake by human breast cancer cells by up to 13-fold when using PEG liposomes compared to PEG-lacking liposomes [190].

However, despite this advantage of the PEG coating of liposomes, it has been reported that after multiple administrations of PEG-coated liposomes, activation of the immune response will begin in the form of the secretion of anti-PEG antibodies. These antibodies will recognize and bind to the liposomes’ surface, resulting in rapid clearance. This phenomenon was referred later to as the “accelerated blood clearance” (ABR) phenomenon [178,179].

Regarding the immune response, several studies have investigated the use of this delivery system to induce a specific immune response [178]. For example, Kawai et al. have developed magnetite cationic liposomes (MCLs) to induce intracellular hyperthermia to treat prostate cancer [191]. In their work, MCL-injected tumour tissues of rats injected with MCLs induced an immune response in which CD3, CD4^+^, and CD8^+^ lymphocytes were detected in the tumour tissues of the treated rats but were not detected in the tumours of the untreated rats [191]. In a study that evaluated the immune response that might be generated after administering TiO_2_ NPs, Auttachoat et al. reported that skin irritation was noticed after dermal administration of TiO_2_ NPs for three days and was consistent with hypersensitivity or inflammatory responses [176]. Moreover, the oral administration of TiO_2_ NPs was reported in another study to increase the activation of mast cells in rats stomachs’ [185]. Carbon-based NPs are considered one type of drug delivery system currently under investigation. This type of NPs includes SWCNTs, MWCNTs, and fullerene C60. The increased use of these NPs was associated with heightened concerns about their immune response. After their dermal administration, SWCNTs increased dermal accumulation of neutrophils and mast cells. This was associated with an increased thickness of the epidermal layer and fibroblast activation [192]. MWCNTs were also associated with the induction of immune responses. The pulmonary administration of these NPs by oropharyngeal aspiration resulted in the release of IL-33, which was detected in the bronchoalveolar lavage fluid. IL-33 release is one of the mast cell activation mechanisms in the lungs, which will release various pro-inflammatory cytokines that cause pulmonary inflammation and fibrosis [193]. Other studies reported other immune responses following MWCNTs administration, such as COX-2 production through a MAPK-dependent mechanism, increased lung inflammation, and increased cytokine releases such as IL-1β, IL-6, and IL-8 production in CD4^+^ and CD8^+^ T cells [176].

The physicochemical properties of specific NPs were shown to play an essential role in the type of immune response elicited by the NP delivery systems. Niikura et al. reported that the shape of AuNP would affect the production of different antibodies and cytokines. Compared to rod-shaped AuNPs, which resulted in half antibody level inflammasome activation and the release of IL-1 and IL-18 release, spherical AuNPs produced the highest antibody level and induced the release of TNF-, IL-6, IL-12, and GM-CSF [194]. Regarding multifunctional mesoporous silica nanoparticles (MSN) and their immune response, Heidegger et al. investigated the immune response of colloidal MSN using mammalian primary murine splenocytes. They demonstrated that the MSN was non-toxic and non-inflammagenic, resulting in trace immune activation. They showed that the MSN was not detected and taken up by T and B lymphocytes, indicating that they lack an immune response to their MSN [195].

## 6. Nanomaterials and Inflammation

Biomaterials are commonly known as foreign substances that cause various inflammatory responses. NP’s inflammatory reaction is based on particle size. For example, inflammation induced by polyacrylic acid coating AuNPs was reported to be based on their hydrodynamic diameters [196]. With a size smaller than 20 nm, fibrinogen-mediated stimulation of the Mac-1 receptor in monocytes in vitro was observed to increase. Mac-1 receptor activation was missing when NPs of 20 nm or larger were used in the monocyte, activating the NF-kB pathway and upregulating other downstream inflammatory cytokines. In vitro, the impact of NP size has also been calculated on inflammatory responses. In poly (d, L-lactide-coglycolic acid) (PLGA), for example, polymorphonucleocyte (PMN) recruitments have proven to be size dependent [197]. Furthermore, NPs with a size of 75 nm elicited minimal PMN recruitment for bronchial alveolar wash material compared to 200 nm particles. The shape is a determinant of inflammatory reactions with respect to the properties of the size. Cellular absorption of rudder-formed NPs more than 100 nm, for example, compared to cones, cubic and cylindrical particles, was greater [198]. In reference, NPs less than 100 nm with spherical geometries were taken up by cells more readily than nanorods or nanosheets [198].

The protein associations at the cell surface cause allergic responses to implanted nanomaterials. The adsorption of proteins, including fibronectin, albumin, vitronectin, and fibrinogen, contributes to various levels of inflammation [199,200]. Nanomaterial composition, charge, and geometrical arrangements are deemed to influence the immune system’s plasma protein adsorption and extracellular matrix components [201]. These cells produce ROS, IL-4, and IL-13, which trigger bloodstream oxidative stress and monocyte recruitment. Implanted biomaterials promote the development of multiproteins that mediate pro-inflammatory cytokines such as IL-1β and IL-18 [202,203]. Monocytes and macrophages that reside in the recruitment develop chemoattraction proteins (GM-CSF, MCP-1, and platelet-dependent growth factor, PDGF) that stimulate colony stimulation (GM-CSF). Such cells often enhance cell-surface expression through Mac-1 receptors and other integrins, which help migrate and bind to surface-adsorbed proteins. Integrin involvement in the production and organization of focal adhesion proteins such as vinculin, paxillin, and α-actinin by adsorbed proteins. Changes in the shape of focal adhesives are converted into cytoskeletal recovery, such as focal adhesive kinase activation (FAK) and signal-driven extracellular kinase (ERK) activation. As described already, FBGCs may shape macrophages under the FBR. Cell fusion of macrophages is induced by the production of IL-4 and Rac-1-mediated, resulting in the production of FBGC that evoke a host of factors such as intermediates of degraded reactive oxygen, remodeled proteins such as MAM and TIMP, TNF-α and other ILs, and the transforming growth factor-substantiation tissue factor. The development and release of macrophages and FBGCs are accompanied by prolonged inflammation, preceded by the penetration, and output of fibroblasts. Development affects the fusion of tissues and implants [204,205].

### Immune Evasion

As an example, if the goal of NPs is to eradicate brain tumor cells, the immune system’s unexpected elimination must be averted. However, if the immune system and immune cells are the intended recipients (as in the case of vaccine administration), NPs must be constructed accordingly [206]. Grafting PEG onto the surface of the particles is the most popular technique to prevent nonspecific elimination by the reticuloendothelial system, thus achieving prolonged circulation duration. This decreases protein adsorption on NPs but does not eliminate it. PEGylation is commonly recommended to increase the stealth capabilities of NPs. However, such polymeric materials are not biodegradable, which may restrict their application, especially if repetitive or chronic distribution is anticipated. Moreover, additional dosages may be cleared more quickly if anti-PEG antibodies are developed [207]. The findings presented by Schöttler et al. indicated that the cellular absorption of polymer-modified NPs might be inhibited by the adsorption of certain proteins. Consequently, the researchers employed PEG-modified polystyrene NPs to confirm, by mass spectrometry, whether the NPs subjected to human plasma contained an abundance of clusterin proteins. By incubating polymer-modified NPs with clusterin, researchers were able to decrease their absorption by the mouse macrophage-like cell line RAW264.7 without compromising the NPs’ biocompatibility. Bertrand et al. discovered that adding clusterin or ApoJ to polymer-based NPs with significant amounts of PEG concentrations did not significantly change the period for which the NPs remained in the blood after being administered intravenously. When NPs encounter blood, there is also a chance that proteins on the surface of the NPs will fall off. The findings presented by Schöttler et al. indicated that the cellular absorption of polymer-modified NPs might be inhibited by the adsorption of certain proteins. Consequently, the researchers employed PEG-modified polystyrene NPs to confirm, via mass spectrometry, whether the NPs subjected to human plasma contained an abundance of clusterin proteins. Researchers were able to stop the mouse macrophage-like cell line RAW264.7 from absorbing polymer-modified NPs by mixing them with clusterin. This did not hurt its ability to be absorbed by the body. The creation of a bio-corona is a continuing dynamic phenomenon, and additional research is required to decipher its biological meaning. It has been noted before how crucial it is to find a happy medium between being taken up by certain target cells and being avoided by immunological or reticuloendothelial phagocytic cells. As a result, there is no practical use for perfect stealth [208]. NPs could be engineered to evade the body’s immune system of the body until they reach their target, such as a solid tumor, where they may be activated locally. Lately, Qiao et al. succeeded in creating a programmable nanotherapeutic that releases latent cytotoxicity against tumor cells by shedding its PEG shell in the tumor microenvironment in a pH-dependent mechanism. Animals given the treatment showed no signs of systemic toxicity after only a few days. Therefore, the results of this investigation provide evidence that NPs may be engineered to achieve both immune evasion and controlled toxicity [209]. Wrapping NPs with PEG is a noninvasive approach to minimizing protein adsorption and minimizing elimination; nevertheless, as described in a previous paragraph, this may also restrict NP absorption at the targeted region; moreover, the polymers could be immunogenic. To bypass these issues, scientists have exploited natural “don’t eat me” signals, including CD47, to endow NPs with proactive stealth capabilities [210]. All cell membranes exhibit the putative “self-marker” CD47. Absorption of red blood cells by macrophages is inhibited by the association of CD47 with CD172a, also known as a signal regulatory protein in phagocytes. Surprisingly, leukemic stem cells upregulate CD47 expression, which increases their pathogenicity [211]. Rodriguez et al. modified polystyrene NPs by computationally constructing minimal “self” peptides from human CD47 to NPs and demonstrating that the self-peptides slowed NP clearance by macrophages in mice engineered to express a modified variant sequence compatible with human CD47. This increased the NPs’ half-life and hence their ability to deliver drugs to xenografts of lung cancer [212]. Nanomicelles of poly(lactide-glycolide)-PEG with stealth capabilities were created by Zhang et al. as an innovative therapeutic and diagnostic platform for simultaneous bioimaging and drug administration in mice with sarcoma, leveraging on the discovery of the minimal “self” peptide generated from the CD47 sequence [213].

## 7. Conclusions

Multiple immunological endpoints have been demonstrated to result from nanoparticle interactions with components of the innate immune system. There is a lack of understanding of these interactions because of their rapidity, complexity, and obscurity. It is well documented that the shape, size, hydrophobicity, and surface functionalization of NPs are critical in controlling their associations with serum proteins and immune cells, particularly APCs. Research has shown that nanotechnology provides several benefits, including increased stability, attractive cellular uptake patterns, delayed drug release dynamics, decreased immunotoxicity, and tailored targeting to specific cell types. Understanding how immunomodulation can be desirable or undesirable due to the route of administration and particle biodistribution in the body—for example, complement activation after intravenous administration is not desirable, but it is beneficial for vaccinations following subcutaneous administration—requires a thorough understanding of the administration route and particle biodistribution in the body. Many concerns remain unanswered about how nanoparticles interact with immune system components, despite recent advances in our knowledge of this interaction. To better understand the physicochemical properties of nanoparticles that define their effects on the immune system, more mechanistic research into particle immunomodulatory effects as immunostimulatory or immunosuppressive potential is required. Although NP cytotoxicity is effective in recognizing acute host injury, it is not sufficiently sensitive to recognize less severe effects or immune system dysregulation. It is also unclear how NPs influence the innate immune system, including the underlying molecular mechanisms. That is why researchers need to put in more time and effort to deduce how NPs influence the immune system so that they can create better techniques for disease treatment and prevention. This review of the available research lends credence to the notion that exposure to nanomaterials, regardless of exposure method, type, size, or solubility, results in a redistribution of the particles to some extent in secondary organs throughout the body.

## Figures and Tables

**Table 1:** Immune system modulation by NPs.

NPs Effect	Immunosuppression	Immunostimulant	References
Desirable	Reducing allergic response	Used as cancer immunotherapy	[27,28]
	Organ and tissue transplant rejection	Enhanced vaccine efficacy	[29]
	Very useful for autoimmune disorders and inflammation treatment	Antitumorigenic	[30]
Undesirable	Severe suppression of the immune system to a level affecting the recognition	Development of inflammation	[31]
	Myelosuppression (bone marrow and thymus gland)	Development of hypersensitivity	[32]
		Development of anaphylaxis	[27]

**Table 3:** Summary of the discussed nanomaterial with their formulation, nature, composition, and their reported medical use.

	Nanomaterial	Medical Use	References
Organic	Chitosan nanosystems	Dental medicine applications	[97,98,99,100,101]
		Wound dressing, tissue regeneration	[48,99,102]
	Silk fibroin	Dental medicine applications	[103,104,105]
		Drug delivery	[106,107,108]
	Graphene- and Graphene oxide- based nanosystems	Gene and small molecular drug delivery Biofunctionalization of proteins Anticancer therapy Antimicrobial agent for bone and teeth implantation Regenerative medicine	[109,110,111] [112,113,114,115,116]
	Fullerenes	Anticancer-targeted drug delivery Antioxidant Anti-infective agents Medical implants, tissue engineering, wound healing, biosensing, bioimaging, vaccination, and photodynamic therapy	[117,118,119,120,121,122,123,124,125,126,127,128]
	Carbon nanowires and nantotubes	Medical implants, tissue engineering, wound healing, chemosensing, biosensing, bioimaging, vaccination, and photodynamic therapy	[128,129,130,131,132]
Inorganic	Metals-based nanosystems	Antimicrobial agents (Ag, Cu, WC, Au, Fe nanosystems)	[133,134,135,136,137,138]
		Targeted drug delivery systems	[139,140,141,142]
		Diagnosis and anticancer therapy (Au, Ag, Zn, Ti)	[143,144,145,146]
	Metal-oxide nanosystems	Antimicrobial agents (TiO2, ZnO, NO, Ag2O, CaO, MgO, CuO)	[135,137,138,147]
		Biosensing and bioimaging	[148,149]
	Dendrimers	Regenerative medicine	[150,151]
		Drug delivery	[146,152,153,154]
		Antimicrobial agents	[155,156,157]

## Data Availability

Not applicable.
